# Experimental Test of Preferences for an Invasive Prey by an Endangered Predator: Implications for Conservation

**DOI:** 10.1371/journal.pone.0165427

**Published:** 2016-11-09

**Authors:** Rebecca C. Wilcox, Robert J. Fletcher

**Affiliations:** University of Florida, Department of Wildlife Ecology and Conservation, 110 Newins-Zeigler Hall, Gainesville, FL, 32611, United States of America; Institut Sophia Agrobiotech, FRANCE

## Abstract

Identifying impacts of exotic species on native populations is central to ecology and conservation. Although the effects of exotic predators on native prey have received much attention, the role of exotic prey on native predators is poorly understood. Determining if native predators actively prefer invasive prey over native prey has implications for interpreting invasion impacts, identifying the presence of evolutionary traps, and predator persistence. One of the world’s most invasive species, *Pomacea maculata*, has recently established in portions of the endangered Everglade snail kite’s (*Rostrhamus sociabilis plumbeus*) geographic range. Although these exotic snails could provide additional prey resources, they are typically much larger than the native snail, which can lead to lower foraging success and the potential for diminished energetic benefits in comparison to native snails. Nonetheless, snail kites frequently forage on exotic snails. We used choice experiments to evaluate snail kite foraging preference in relation to exotic species and snail size. We found that snail kites do not show a preference for native or exotic snails. Rather, snail kites generally showed a preference for medium-sized snails, the sizes reflective of large native snails. These results suggest that while snail kites frequently forage on exotic snails in the wild, this behavior is likely driven simply by the abundance of exotic snails rather than snail kites preferring exotics. This lack of preference offers insights to hypotheses regarding effects of exotic species, guidance regarding habitat and invasive species management, and illustrates how native-exotic relationships can be misleading in the absence of experimental tests of such interactions.

## Introduction

Invasive species are an increasingly common part of modern ecosystems. Invasive species can have profound negative effects on native species through competition for resources and predation. However, they also have the potential to facilitate native species through habitat modification or increased resources [1]. The implications of these interactions become intensified for native species that might be at greater risk of extinction due to specialized resource needs [2]. In such cases, invasive species can be deadly [3] or present a unique opportunity to provide critical services, such as nesting habitat, refugia, or increased prey resources in native systems that have otherwise become degraded [4, 5].

Invasive species may impact native species by influencing resource preference of native species. Schlaepfer et al. [6] suggested that a major impact of invasive species is that they could trigger evolutionary traps for native species, when individuals prefer resources that are associated with decreased fitness benefits [7]. This issue is particularly important for endangered species because evolutionary traps can facilitate extinction (e.g., [8, 9]). An important component for understanding if an evolutionary trap exists is to determine whether native species may prefer novel exotic species that invade ecosystems. Preferences for invasives can arise through preferences in habitat, such as nesting substrate [4], mating preference [10], or foraging preferences [11, 12].

Foraging preference of native predators selecting between native and invasive prey may have implications for consumer-resource interactions, predator-mediated indirect interactions [13], invasive species success, as well as predator distribution and persistence [14]. However, reliably estimating predator preference in wild populations can be challenging [7]. Choice experiments provide causal inference on preference, allow the identification of cues that predators use to select prey, and can help determine the consistency of preferences across space [15, 16]. Despite the benefits of choice experiments, they are infrequently used for assessing preference in wild vertebrate populations in comparison to use-availability designs [7]. This is due to the difficulty of performing controlled experiments with wild individuals of many species. However, when choice experiments can be implemented, they allow for control of the density of prey available to the consumer. In contrast, in use-availability studies it can be difficult (in most systems) to effectively make the link between the density of prey that is present in the environment and what prey is available to the consumer [17].

**Fig 1:**
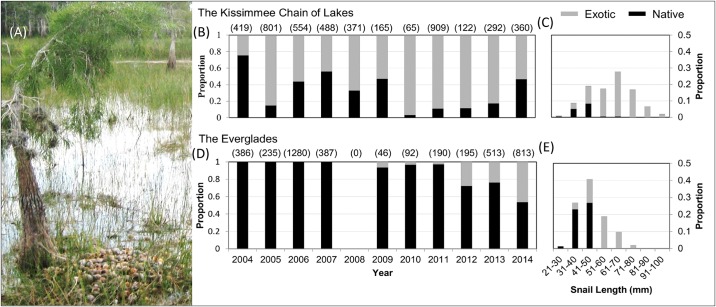
Snail kite consumption of native and exotic snails. The proportion of native (black) and exotic (gray) snails consumed by snail kites between 2004 and 2014 in the (B) Kissimmee Chain of Lakes and the (D) Everglades. Sample sizes are noted in parentheses above each bar. Histograms show the proportion of native (black) and exotic (gray) snails consumed, by size class, in the (C) Kissimmee Chain of Lakes in 2013 (n = 292) and the (E) Everglades in 2014 (n = 813). This species distinction illustrates the expansion of snail sizes made available by the presence of the exotic snail. These data were collected as part of a long-term monitoring program monitoring snail kite demography [21]. During this monitoring effort snail shells were collected opportunistically from snail kite foraging perches within each of the sampling regions. (A) An example of a foraging perch; a cypress tree (*Taxodium distichum*), with a pile of discarded snail sells underneath. Shell length was measured in the same way that snails were for experiments (see Methods). No snail shells were collected in the Everglades in 2008.

We used choice experiments to test for the foraging preference of the endangered Everglade snail kite (*Rostrhamus sociabilis plumbeus*) for native relative to invasive prey. The snail kite is an extreme dietary specialist, historically foraging almost entirely on a single species of native Florida apple snail (*Pomacea paludosa*). An ongoing invasion by a large exotic snail (*Pomacea maculata*), considered to be one of the greatest threats to freshwater ecosystems in the United States [18], is occurring within the kite’s range. The exotic snail often reaches very high abundances in these ecosystems relative to native snails [18], and kites now actively forage on exotic snails (Fig 1). However, it has been hypothesized that exotic snails may be an evolutionary trap for kites because of potential energetic deficits that can arise from foraging difficulties due to their large size, particularly for young birds (≤ 1 year old), and the assumption that kites may prefer exotics given that they frequently consume them [19]. Because first-year survival is a limiting factor for snail kite population growth [20], being able to interpret the potential for an evolutionary trap will provide sound guidance for conservation of this endangered bird. However, first it is important to understand whether kites actively prefer exotic snails or are simply responding to the high abundance of exotic prey.

Given the importance of prey type for snail kite energetics [16] and the potential negative impact that large exotic snails have on daily time budgets [19], we expected that variation in foraging preference by snail kites could be driven by species, snail size, or prey profitability (total energy content divided by its handing time). Because native and exotic snails are similar in appearance (other than size), we expected that snail kites (a visual predator) would show no preference for either species once size was controlled for in the experimental design. In contrast, we expected that snail kites would have foraging preferences based on either size or profitability of snails. For instance, Beissinger et al. [16] considered the role of prey size and profitability for snail kites that were selecting between snails and crabs in South America. In addition, since the invasion has occurred at different times in different sites across the region, we expected their preference may vary based on invasion history, either due to variation in experience or variation in general numbers and sizes of snails that may occur locally. Here, we used choice experiments to determine if kites show a preference for (1) exotic or native prey, (2) show a preference for exotic snails based on snail size, and (3) assessed how consistent preferences are across individuals and between regions that have experienced different invasion histories of the exotic snail.

## Materials and Methods

### Study system

The snail kite is a wetland-dependent raptor whose range is restricted to a network of wetlands in central and southern peninsular Florida [21]. We conducted field experiments of foraging preference in two primary regions within the kite’s range in 2013 and 2014: the Kissimmee Chain of Lakes and Water Conservation Area 3A. The Kissimmee Chain of Lakes is located in the northern portion of the kite’s range and has historically been considered kite refugia habitat [22]. In this region, kites use littoral habitats along the fringes of lakes (lacustrine habitats dominated by cattail (*Typha* spp.) and bulrush (*Scirpus* spp.)) for foraging and reproduction. Water Conservation Area 3A is located in the southern portion of the kite’s range, and is part of the Everglades, which has historically been of great importance for kite reproduction [19] (hereafter the Everglades). The Everglades consist of broad expanses of freshwater marsh (palustrine) habitat dominated by sawgrass (*Cladium jamaicense*).

We selected these areas for three reasons. First, they are historically and currently areas that have high snail kite reproduction, such that they are areas considered to be critical for population recovery. Second, both areas are highly managed for recreation and conservation, such that management decisions regarding invasive species control and snail kites are complex in the regions. Third, these areas are at the extremes of the range (approximately 200–270 km apart), and dispersal, both within breeding seasons and between consecutive breeding seasons, is relatively infrequent between these two areas [23], particularly for the time period being considered here. Limited movement between these areas gives us confidence that we would not likely resample individuals between sites during experimental trials.

While the native and invasive snails are quite similar in appearance, the exotic snail grows much larger, has a higher drought tolerance, lives longer, produces more eggs per egg mass, and can occur in much higher densities [18, 24, 19]. In recent years, the native snails are thought to have declined within the snail kite’s range [25]. The exotic snail first became established in the Kissimmee Chain of Lakes in 2004 [26]. Exotic snails have only been observed more recently and in limited areas in the Everglades (Fig 1). Consequently, these two regions not only vary in the type of wetland habitat but also in the invasion history of the exotic snail. The invasion history is important given that it may influence the abundance of snails in the area and the size distribution of snails available to kites (Fig 1).

### Ethical Statement

This study was approved and conducted under the University of Florida Institutional Animal Care and Use Committee permit no. 201005469. The lakes/wetlands visited within the Kissimmee Chain of Lakes (centroid of sampling locations: Lake Kissimmee 465505, 3117712 UTM, East Lake Tohopekaliga 473044, 3126167, Lake Cypress 465986, 3104885 UTM, Lake Hatchineha 463652, 3001583, Lake Tohopekaliga 459867, 3126989 UTM) and Water Conservation Area 3A (centroid of sampling locations: 520916, 2850564 UTM) are public lands managed by the Florida Fish and Wildlife Conservation Commission that do not require a permit to access.

### Experimental design

Foraging preference commonly refers to a non-random selection of prey types (e.g., species, size, etc.) by predators when prey types are presented in equal amounts [27]. Foraging preference is often assessed in the field with use-availability designs (e.g., [28]), but in many systems determining what is not only abundant in the system but also available to the consumer can be difficult [17]. While choice experiments present a semi-artificial environment, they allow us control the abundance and availability of prey locally to isolate potential foraging preferences.

To address our questions, we used two different choice experiments, one focused on testing for species preference and a second testing for size preference. We considered snail size because previous studies on foraging kites focused on the relationship between size and handling time and its implications for energetics [16], and exotic snails are generally—but not always (Fig 1)—larger than native snails. For the first experiment, we tested the effect of species on choice by setting out two trays, one held native snails and the other exotic snails, where we attempted to pair natives and exotics of similar size. For the second experiment, we determined effects of snail size by setting out two trays that both held exotic snails only but the size of snails varied between trays. For the latter experiment we used only exotic snails to control for any potential species effect and because the exotic snails spanned the entire gradient of snail size that kites have been observed to consume in the region (Fig 1).

**Fig 2:**
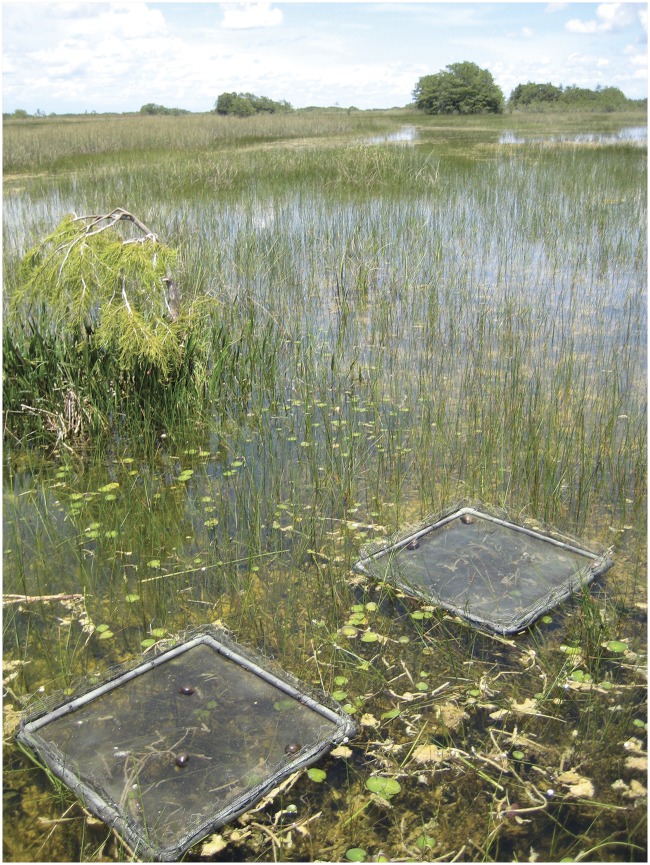
Choice experiment trial setup. An example of a two-tray choice experiment trial placed next to a common snail kite foraging perch (*Taxodium distichum*).

Choice experiment trials took place by setting 2 trays 1–3 meters from a kite foraging perch (Fig 2). Individuals will often habituate to foraging perches, frequently using them for extracting snails (Fig 1A). These perches include natural (e.g. trees and shrubs) and man-made perches (e.g. fence posts and artificial platforms; [29]) that provide stable support for extracting snails. These perches can be identified by large piles of snail shells (e.g., 10–100 shells) that accumulate on or below these perches, implying frequent use. Foraging perches are often used to evaluate what sizes of snails that kites consume in a given area (e.g. [30–32]).

Kites are visual hunters that use both perch hunting and course hunting techniques [31]. As in Beissinger et al. [16], we focus on perch-hunting birds. By using trays that positioned snails just below the water’s surface, we controlled for any effect that water clarity and vegetation obstruction might have on choice and ensured that individuals would be able to view all prey options before making a choice, thus isolating potential preferences from habitat confounding availability to individuals. For each trial, we attempted to test a different individual. To do so, we (1) performed trials primarily during the breeding season (89% in June-August, 11% in September) when kites congregate in breeding areas and are less likely to move between sites than during the non-breeding season [23]; (2) performed trials > 200 m apart; and (3) we used plumage characteristics to ensure that nearby trials within sites were on different individuals.

The range of snail sizes used in choice experiments was limited by what could be collected from the wild in each region: 30–90 mm in the Kissimmee Chain of Lakes and 40–70 mm in the Everglades. All snails were given a unique label, identified to species and measured for length. Length was measured linearly along the spiral axis of the snail shell from the apex to the outer edge of the aperture lip [19]. Four live snails were placed in each 1 m^2^ mesh lined PVC tray of the same species and roughly the same length (+/-7 mm) to simulate high food density when animals are hypothesized to be most selective [33]. The perimeter of each tray was lined with a 6 cm high wire fence to prevent snails from escaping. Both experiments were conducted in each study area with individuals that ranged in age (juvenile, adult), sex (male, female, unknown) and breeding status (breeding, non-breeding). Juveniles were considered to be individuals that are less than 6 months old; they have distinct eye color and plumage [34]. Adults were considered any individual over 6 months old. Snail kites are sexually dimorphic but do not reach distinct plumage characteristics between the sexes until 36 months [34]. For this analysis individuals were divided into 3 classes: male, female and unidentified. Individuals were considered breeding if they were associated with an active nest [35].

Experimental trials lasted until an individual made four choices or had been at the perch for one hour. Once trays were set, we conducted observations of foraging choice and handling time either in person (91% of trials) or recorded them with a GoPro (9% of trials; GoPro Hero 3) camera for later review. We conducted 64 experimental trials: 25 trials of native and exotic snail comparisons and 39 exotic-only trials. Thirty-four trials were conducted in the Kissimmee Chain of Lakes in 2013 and 30 in the Everglades in 2014.

### Analysis

To evaluate factors affecting foraging preference in addition to snail species and size, we also considered prey profitability [16]. Profitability can be an important metric because it combines both the caloric content of the prey and the consumer’s ability to handle the prey. Profitability, *p*, of snail *i* consumed was calculated as:

$$p_{i}=g_{i}\times \frac{cal_{i}}{t_{i}}$$ (1)

where *g* is the dry eatable weight (grams), *cal* is the caloric content (kcal/g), and *t* is handling time in seconds [16, 19]. Dry eatable weight was calculated from wet weight eatable tissue, estimated from shell length using linear regressions calculated in Cattau et al. [19] for exotic snails (*R*^2^ = 0.75) and in Sykes [31] for native snails (*R*^2^ = 0.75). We used caloric content for native (4.60 ± 0.18 kcal/g) and exotic snails (3.25 ± 0.11 kcal/g) from Sykes [31] and Cattau et al. [19] respectively, who both sampled snails from within the snail kite’s range. Handling time *t* was calculated for each snail consumption event in trials during observations as, once perched, the amount of time it took for an individual to extract the snail from the shell, remove uneatable parts (reproductive organs in female snails) and then consume the meat [31, 16].

To appropriately capture the paired design of experiments, we used a conditional logistic regression to estimate kite foraging preference using the survival package in program R and considered each experimental trial as a stratum [36]. Three groups of model selection comparisons were conducted to determine: (1) if kites show a preference for species; (2) what characteristics of exotic prey drive preference (size or profitability); and (3) the role of consumer characteristics and region on choice. First, we used the data from the first experiment (trays that compared native and exotic snails) to determine if snail species, size or profitability explained variation in choice. We attempted to control for size during experiments (< 30 mm difference in size) but due to high correlation between size and species in the sample (*r* = -0.78), species and size could not be included in the same model. Because estimated profitability is partially a function of size (Eq 1), size and profitability were highly correlated (*r* = 0.97) and were also not included in the same model because of their high correlation. Note, however, that we expected generally positive effects of profitability with preference, while we expected that size may have non-linear relationships with preference (see below). Second, we used data from the second experiment (trays of exotic snails that varied in size) to determine if snail size or profitability better explained foraging choices. For these first two groups of models, we considered linear and non-linear (quadratic) models for snail size and a linear model for profitability. Linear relationships between prey size and selection can occur in the wild given natural variations in prey [37] but given the large range of sizes presented by native and exotic snails (30–90 mm) and the previously reported difficulty of kites handling large exotic snails [38, 19], it is possible that a limit for the ideal size range should exist [39], such that a non-linear (e.g., quadratic) relationship may occur. Third, we pooled data from both experiments to assess how consistent size preferences are across individual characteristics (sex, age, breeding status) and regions. To determine if region, individual age, sex or breeding status could mediate potential preferences for snail size, we added appropriate interactions to each model in the third set of comparisons. Note that main effects for these factors (region, age, sex, breeding status) were not included because in this study design, these factors did not vary within trials, such that main effects are not estimable (see, e.g., [40]).

We performed the analysis in two ways. First, we defined foraging preference of kites based on the first choice, when prey options were available in equal amounts [26]. Second, we used a conditional logistic regression that accounted for repeated measures (via generalized estimating equations) to evaluate the effect of prior knowledge on preference based on all choices made during a trial. This allowed us to understand variability in preference through multiple choices, which would imply that the first choice might be exploratory rather than preferential. Models were evaluated with Akaike’s Information Criterion accounting for small sample size (AICc) and AICc weights [41]. The models that had the lowest AICc value and the highest AICc weight were considered to be top performing models [41].

## Results

**Fig 3:**
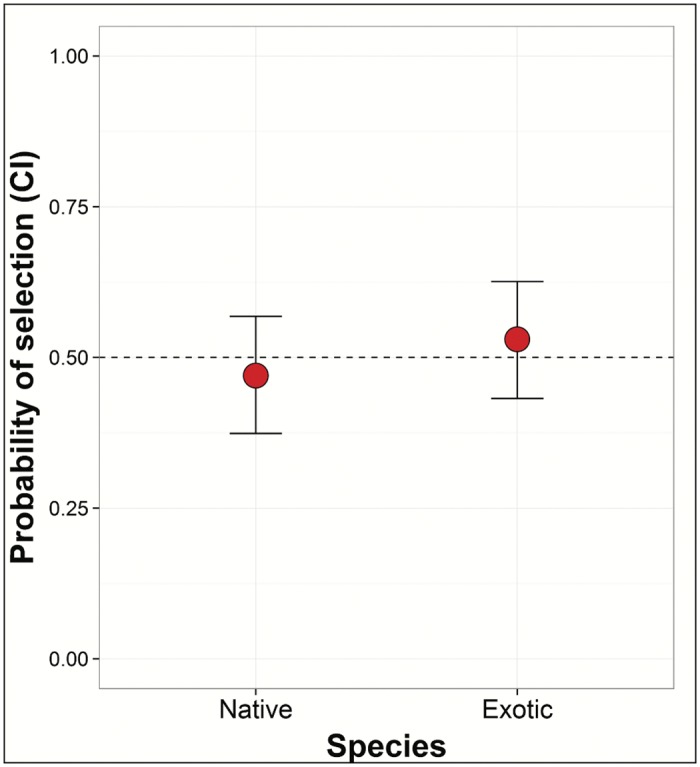
Probability of snail kite selecting a native or exotic snail. Model predicted foraging preference (with 95% confidence intervals) of snail kites foraging on native or exotic snails using only the first choice made during experimental trials.

Based on the first choice made during trials, when selecting between native and exotic snails, kites chose native snails 13 times (mean size = 39.23 mm) and exotic snails 12 times (mean size = 53.40 mm). Model selection suggested that snail size drove selection, with the top two models including size and a combined 72% of AICc weight (Table 1), although the 95% confidence intervals around the parameter estimates for the top model slightly overlapped zero (β_size_ = 0.54, 95% CI = -0.03–1.12, β_size2_ = -0.005, 95% CI = -0.01–0.0001). While the model for snail species received some support (0.12 AICc weight), the confidence intervals of the parameter estimate for species substantially overlapped zero (β_species_ = -0.24, 95% CI = -1.03–0.55), suggesting little overall support for the effect of snail species on choice (Fig 3).

**Table 1:** Results of conditional logistic regression testing for the effects of various factors on preference using the first choice made during trials.

Model	K	LL	AIC_c_	*Δ* AIC_c_	AIC_c_ Weight
Choice between native and exotic snails (n = 25) ^a^					
size + size^2^	2	-14.58	33.41	0.00	0.54
size	1	-16.77	35.60	2.22	0.18
species	1	-17.15	36.38	2.97	0.12
profitability	1	-17.23	36.54	3.13	0.11
profitability + species	2	-17.14	38.50	5.08	0.04
Exotic snail choice (n = 39) ^b^					
size + size^2^	2	-22.83	49.80	0.00	0.90
profitability	1	-26.71	55.50	5.65	0.05
size	1	-26.83	55.70	5.88	0.05
Individual traits and region (n = 64) ^c^					
size + size^2^ + size × region	3	-34.33	74.85	0.00	0.94
size + size^2^	2	-39.05	82.20	7.35	0.02
size + size^2^ + size × age	3	-38.17	82.50	7.65	0.02
size + size^2^ + size × breeding	3	-38.63	83.50	8.65	0.01
size + size^2^ + size × sex	3	-38.03	84.37	9.52	0.01

K is the number of parameters in the model, LL is the model likelihood, AICc is Akaike’s Information Criterion adjusted for small sample size, ***Δ***AIC is the difference in AICc from one model to the best ranked model and AICc Weight is a normalized representation of the model likelihoods so that they are treated as relative probabilities for comparison [41].

^a^ Individuals selecting between native and exotic snails.

^b^ Individuals selecting between exotic snails of differing sizes.

^c^ The role of age, sex, breeding status and region in explaining variation in preference for snail size.

**Fig 4:**
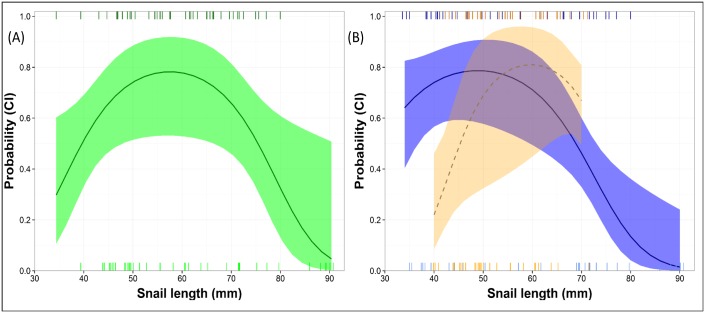
Probability of snail kite foraging selection by snail size. Model predicted foraging preference (with 95% confidence intervals) using only the first choice made during trials. (A) Snail kites showed a high probability of selecting snails between 50 and 65 mm in length. Sizes of snails selected during trials are represented by dark green dashes on top of plot and snails that were not selected are represented by light green dashes on the bottom of the plot. (B) Snail kites in the Kissimmee Chain of Lakes (model prediction is the solid line with blue CI, dark blue dashes represent snail sizes that were selected and light blue dashes represent snails that were not selected) showed a higher relative probability of selecting smaller sized snails (40–55 mm) than those individuals in the Everglades (model prediction is the dash line with orange CI, dark orange dashes represent snails that were selected, light orange dashes represent snails that were not selected).

When considering trials with exotic snails only to examine the effect of size on choice, the model including the quadratic term for snail size had the most support (Table 1). Point estimates were similar to those from the native-exotic experiment, yet confidence intervals for the parameter estimate for this model did not overlap zero (β_size_ = 0.45, 95% CI = 0.04–0.88, β_size2_ = -0.004, 95% CI = -0.008 − -0.0004). The average snail chosen during these trials was 59.12 mm in length and model predictions suggested that kites foraging on exotic snails tended to prefer medium-sized snails between 50 and 65 mm in length (Fig 4A). When examining the role of individual characteristics and region to further explain variation in size selection, the model that included the interactive effect of region on size showed the most support in model selection (β_size × region_ = 0.10, 95% CI = 0.03–0.17, Table 1). Kites in the Kissimmee Chain of Lakes showed a higher relative preference for snails between 40 and 55 mm in length than did kites in the Everglades (Fig 4B). We found no evidence for age, sex, or breeding status influencing size selection (Table 1).

We then evaluated the role of factors affecting the variation in preference when individuals make multiple choices. Results from model selection were similar to those found when evaluating just the first choice (Table 2), suggesting that changes in choices within trials were not occurring and that the first choice was an accurate representation of foraging preference.

**Table 2:** Results of conditional logistic regression testing for the effects of various factors on preference using all choices made during a trial.

Model	K	LL	AIC_c_	*Δ*AIC_c_	AIC_c_ Weight
Choice between native and exotic snails (n = 25) ^a^					
size + size^2^	2	-102.47	209.00	0.00	0.53
size	1	-103.68	209.40	0.37	0.44
profitability	1	-106.97	216.00	6.95	0.02
species	1	-107.17	216.40	7.35	0.01
profitability + species	2	-106.96	218.00	8.99	0.01
Exotic snail choice (n = 39) ^b^					
size + size^2^	2	-106.26	216.60	0.00	0.79
size	1	-108.91	219.90	3.25	0.16
profitability	1	-109.91	221.90	5.25	0.06
Individual traits and region (n = 64) ^c^					
size + size^2^ + size × region	3	-205.28	416.60	0.00	0.998
size + size^2^	2	-213.24	430.50	13.88	0.001
size + size^2^ + size × age	3	-212.86	431.80	15.17	0.001
size + size^2^ + size × breeding	3	-213.15	432.40	15.74	0.000
size + size^2^ + size × sex	3	-212.74	433.60	16.98	0.000

K is the number of parameters in the model, LL is the model likelihood, AICc is Akaike’s Information Criterion adjusted for small sample size, ***Δ***AIC is the difference in AICc from one model to the best ranked model and AICc Weight is a normalized representation of the model likelihoods so that they are treated as relative probabilities for comparison [41].

^a^ Individuals selecting between native and exotic snails.

^b^ Individuals selecting between exotic snails of differing sizes.

^c^ The role of age, sex, breeding status and region in explaining variation in preference for snail size.

## Discussion

Exotic prey are hypothesized to have a variety of effects on native predators, such as negative effects by potentially causing evolutionary traps or positive effects by potentially providing supplemental resources [6, 5], which is largely dependent on the extent to which native predators can recognize and potentially prefer exotics. Yet understanding these potential effects has been challenging for several reasons, including the need to have reliable estimates of preference [7]. Our results shed light regarding factors driving the foraging preference of snail kites, a critically endangered raptor, and provide insights for management priorities and the potential role of prey invasion history on predators.

We found little support for preference based on snail species (Fig 3). This lack of preference could be due to similarities in morphology between the two prey species. Search image and nutrient content of prey can affect foraging [42] and in this system snails are very similar in both appearance and nutrient content (content of fat and carbohydrates), but differ greatly in size [19]. Given these similarities, it is unknown if kites show a lack of preference for snail species due to an inability to differentiate them or if they simply do not have a species preference. In any case, our results suggest that kites have not developed a preference for exotic over native snails. Rather, the fact that snail kites will frequently consume exotic snails in the wild (Fig 1) is likely driven simply by the abundance of this particularly fecund invasive species. If such foraging is simply driven by exotic snail abundance, then conservation aimed at restoring native snail abundance may result in a return of snail kites foraging more frequently on native snails [43, 44] and provide better opportunities for management control options of exotic snail populations that minimize impacts on snail kites [26].

Prey size influences foraging preference for visual predators because it can affect encounter rates, capture success, handling time, and energy consumption [45, 19]. Our results complement prior work by showing that for kites, prey size rather than species or profitability, is the cue that consistently explained the most variation in foraging preference. However, the high correlation between size and profitability (see Eq 1) makes it difficult to isolate the role of each. Nevertheless, if kites are responding to profitability, we would expect a positive relationship with this metric (and thus size, due to their correlation), while evidence supports that size has a non-linear relationship (Fig 4). Future work aimed at better isolating these issues would be helpful. Previous studies with predators have found a linear relationship between prey size and selection [37, 16], yet our results showed that kites prey preference peaks at moderate sizes, and kites generally showed a preference for medium-sized snails (50–65 mm; Fig 4B). This result suggests that despite the emergence of a larger size class of snail (>75 mm) presented by this novel exotic species [46], kites continue to prefer snails the size of a large adult native snail, perhaps due to limitations in handling time by bill and talon morphology when consuming larger snails [38, 19, 26].

Region, rather than individual characteristics, explained the most variation in preference for snail size. This result could be due to: (1) local invasion history of native snail; (2) habitat type; or (3) experimental constraints. Native predators may adapt to the presence of invasive prey over time [5]. This hypothesis could explain why region, rather than individual characteristics, explained variation in size preference. The invasion history, density and size distribution of snails differs greatly between the two regions (Fig 1), which could affect an individual’s foraging expectations and preferences [47]. For instance, given that exotic snails have a significantly lower caloric content per g of dry weight than native snails and the handling difficulties observed in foraging on larger snails [38, 19], individuals in areas with a longer invasion history (i.e., the Kissimmee Chain of Lakes) may have learned to prefer smaller prey, while individuals in areas with a shorter invasion history may be more naïve, tending to avoid small prey for and rather prefer this novel-sized large exotic prey. While kites are thought to be nomadic species and likely travel to areas with different prey distributions [48, 23], this result suggests that it could be the more recent foraging experiences that shape current preference rather than longer-term experiences [49]. The difference could also be due to differences in habitat type; however, while habitat could alter snail availability our study design enabled us to control for this issue. Yet habitat could influence foraging behavior in natural settings [50] and could be important at larger scales, such as site (wetland) selection. Alternatively, this regional result could also be due to the study design. Different size classes of snails were offered in each region, based on what was available and collected in the area. However, the experimental analysis contrasted within-trial size variation, such that while we could not assess the entire gradient of variation in the Everglades, there was support for changes in preference based on the availability present in each region (i.e., we observed differences in choices for the size range, 40–70 mm available in both regions; Fig 4B). Further work to understand the extent of these regional differences and how they may change over time would be helpful.

Invasive species are a leading cause of evolutionary traps [6] although oftentimes the presence of traps is misidentified [51]. Robertson et al. [51] emphasized that in order to identify the presence of evolutionary traps correctly, individuals must show a preference for a resource that is associated with reduced fitness. Previous work with kites hypothesized that an evolutionary trap may occur from exotic snails because energetic benefits diminished for juvenile kites foraging on large exotic snails, which could lead to decreased juvenile survival. This hypothesis was also emphasized because kites frequently attempted to forage on this new resource, suggesting potential preference for exotic snails [19]. Our results highlight no preference by kites for snail species and rather a preference for medium rather than large snails (regardless of snail species), providing experimental evidence refuting the possibility of an evolutionary trap in the system. Taken together, these results emphasize that snail kites are not preferring large exotic snails (that cause increased number of drops and handing time; [38, 19]) and may not select them if suitable alternatives are available. By restoring native snails (sizes of which we show kites to prefer) to high abundances, such conservation strategies would provide a native food source for the snail kite population rather than an exotic prey that could have negative impacts on the ecosystem as a whole [18].

While choice experiments provide strong inference on preference [7], there are some limitations with choice experiments and our experimental design. First, the trials were designed to present only two prey options at a time, which may be unlikely to occur in nature. Yet by replicating the experiments with multiple options of snails and different individuals, preference estimation across the population was feasible. Second, trials could not be set up to evaluate choices made while course hunting, which could be different from perch hunting. Finally, the choice experiments implemented here do not test for larger-scale decisions that individuals may make when selecting foraging habitat. Yet despite these limitations, choice experiments allowed us to isolate preference of a wild endangered population and better understand the relationship between snail kites and exotic snails, which would have been unclear in the absence of such experiments.

Previous studies have focused primarily on the prey in interactions between native predators and invasive prey, even though predator response has implications for invasive species success as well as predator distribution, abundance, and persistence [14, 5, 52]. Here we identified consumer preference and the factors that affect resource selection. Identifying the cues that consumers use to select prey can help interpret why predators might forage heavily on invasive species or why they might avoid them. This issue is important given that these choices can have strong demographic consequences on native consumers [53, 44, 26]. In situations where endangered species appear reliant on invasive species [4, 54], management decisions concerning invasive species can become difficult such that understanding precisely the relationships between endangered and invasive species is essential. Accurately identifying resource selection cues by consumers will help guide invasive species management and conservation as invasive species continue to expand and impact native systems.

## Supporting Information

S1 Dataset Choice experiment dataset and metadata. (CSV)
